# Values and preferences in oral anticoagulation in patients with atrial fibrillation, physicians' and patients' perspectives: protocol for a two-phase study

**DOI:** 10.1186/1472-6963-8-221

**Published:** 2008-10-27

**Authors:** Pablo Alonso-Coello, Victor M Montori, Ivan Solà, Holger J Schünemann, Philip J Devereaux, Cathy Charles, Mercè Roura, M Gloria Díaz, Juan Carlos Souto, Rafael Alonso, Sven Oliver, Rafael Ruiz, Blanca Coll-Vinent, Ana Isabel Diez, Ignasi Gich, Gordon Guyatt

**Affiliations:** 1 Iberoamerican Cochrane Center, Hospital Sant Pau, Sant Antoni Maria Claret 171, Barcelona, Spain; 2 CIBER de Epidemiología y Salud Pública (CIBERESP), Spain; 3 Knolwedge and Encounter Research Unit, Mayo Clinic, 200 First Street SW, Rochester, MN 55905, USA; 4 Department of Clinical Epidemiology & Biostatistics, CLARITY Research Group, McMaster University Medical Centre 2C9, 1200 Main St W, Hamilton, ON, Canada; 5 Institut Català de la Salut, Lope de Vega, 138, Barcelona, Spain; 6 Unidad Docente de Medicina Familiar y Comunitaria, Hospital Donostia, Paseo Dr. Beguiristain s/n, Donostia, Spain; 7 Unitat d'Hemostàsia i Trombosi, Hospital Sant Pau, Sant Antoni Maria Claret 171, Barcelona, Spain; 8 Equipo de Atención Primaria Griñón, Area 10, Madrid, Spain; 9 Unidad Formadora de Medicina Familiar y Comunitaria de A Coruña, Spain; 10 Institut Català de la Salut, c/Sardenya 375. Entlo 4a, Barcelona, Spain; 11 Hospital Clínic. Villarroel 170, Barcelona, Spain; 12 Centro de Salud de Beraun. Avenida Galtzaraborda 67, Errenteria, San Sebastian, Spain

## Abstract

**Background:**

Oral anticoagulation prevents strokes in patients with atrial fibrillation but, for reasons that remain unclear, less than 40% of all patients with atrial fibrillation receive warfarin. The literature postulates that patient and clinician preferences may explain this low utilization.

**Design:**

The proposed research seeks to answer the following questions: i) When assessed systematically, do patients' and clinicians' preferences explain the utilization of warfarin to prevent strokes associated with atrial fibrillation? ii) To what extent do patients' and clinicians' treatment preferences differ? iii) What factors explain any differences that exist in treatment preferences between patients and clinicians? To answer these questions we will conduct a two-phase study of patient and clinician preferences for health states and treatments. In the first phase of this study we will conduct structured interviews to determine their treatment preferences for warfarin vs. aspirin to prevent strokes associated with atrial fibrillation using the probability trade-off technique. In the same interview, we will conduct preference-elicitation exercises using the feeling thermometer to identify the utilities that patients place on taking medication (warfarin and aspirin), and on having a mild stroke, a severe stroke, and a major bleed. In the second phase of the study we will convene focus groups of clinicians and patients to explore their answers to the exercises in the first phase.

**Discussion:**

This is a study of patient and clinician preferences for health states and treatments. Because of its clinical importance and our previous work in this area, we will conduct our study in the clinical context of the decision to use antithrombotic agents to reduce the risk of stroke in patients with non-valvular chronic atrial fibrillation

## Background

Chronic atrial fibrillation (AF) is the commonest sustained cardiac arrhythmia. The prevalence of AF increases from less than 1% in patients younger than 60 years to almost 10% in patients over the age of 80 years [1,2]. Similarly, the incidence of AF increases from 0.2% per year in men under the age of 40 years to more than 2% per year in men aged 80–89 years, with a lower age-adjusted incidence in women [3]. This condition is associated with substantial mortality and morbidity from stroke, thromboembolism and heart failure [4]. On average, 5 out of every 100 patients will have a stroke every year of whom 3 will have severe disability or die prematurely [5,6]. Warfarin reduces the risk of strokes by 65% [7].

A recent systematic review of practice surveys found that 56 to 85% of patients with atrial fibrillation are not receiving warfarin [8]. As a result thousands of these patients will suffer preventable strokes every year and many will be left with severe and permanent disability or will die prematurely. Why do these patients and their clinicians choose not to use warfarin? Although this review identifies some system barriers to anticoagulation [8], the most common reason for not offering and prescribing warfarin to patients with atrial fibrillation was clinicians' perception that patients were at high risk of bleeding [9]. This point suggests that clinicians must consider the majority of patients with atrial fibrillation to be at sufficiently high risk of bleeding to warrant withholding warfarin. This behavior is consistent with clinicians being more bleeding-averse than stroke-averse. The catastrophic impact of major stroke on patients' lives raises questions about these apparent values. The wisdom of withholding anticoagulation is further challenged by data suggesting that clinicians' ability to assess their patients' risk of bleeding on warfarin is no better than chance [10].

One needs to weigh the benefits of stroke prevention against the inconvenience and cost of taking warfarin daily, the inconvenience of periodic blood testing to monitor anticoagulation, and the risk of both minor and major bleeding. In addition, there are alternatives to warfarin. Aspirin is less effective than warfarin in avoiding strokes, it is less likely to cause bleeding [11], and requires no laboratory testing to monitor its effects. If patients choose not to take either warfarin or aspirin, they minimize their risk of bleeding and the inconvenience of using these treatments, but are left with an increased risk of stroke.

**Figure 1:**
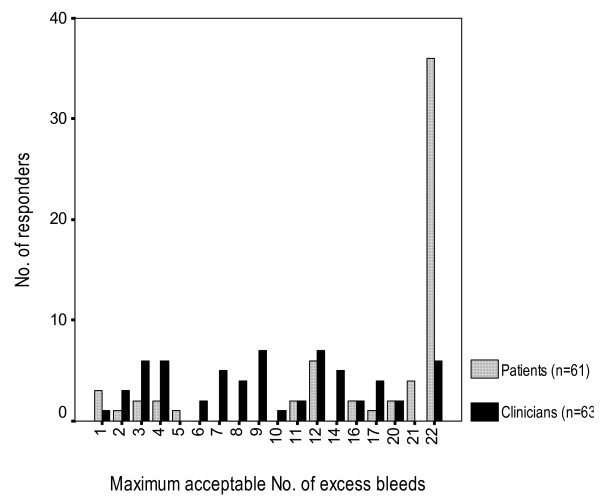
**Bleeding thresholds for warfarin**[12]

Perhaps patients and clinicians do not choose to use warfarin because they prefer to avoid the risk of bleeding associated with its use. This perspective may be particularly true for those patients considered at high risk of bleeding. In this proposal, we will describe a prior study we conducted that suggests that this explanation is unlikely [12]. Our results suggested that patients were much more stroke-averse than bleeding-averse (Figure 1). On the other hand, clinicians were less stroke-averse and more bleeding-averse than the patients. If these results are accurate and widely generalizable, they suggest that warfarin is underutilized and that the utilization rates are not consistent with patient values but are determined by physician values. Another way to look at the results involves determining what participants implied about the relative values of strokes and bleeds. In effect, clinicians' felt that the adverse consequences of a severe gastrointestinal hemorrhage had more or less the same value (or disutility) as the adverse consequences of a stroke. This was in spite of describing that half of the strokes were severe enough to lead to irreversible major disability or death and of describing severe gastrointestinal bleeds as transitory events associated with transfusions, endoscopy, and relatively rapid recovery to pre-morbid function.

If our findings were valid (i.e., they truly reflect individual patient and clinician choices) and generalizable, they would have profound implications. First, they suggest that current clinician behavior of withholding warfarin in patients at risk of bleeding is inconsistent with the values and preferences of patients. Second, if clinicians were to behave in a way consistent with patient preferences, patients would likely experience fewer strokes thanks to wider warfarin use. Thus, if confirmed, our findings will call for interventions to align clinician behavior with patient preferences.

It is possible, however, that other considerations may explain our previous findings. First, our previous study lacked an optimally detailed presentation of warfarin use. To varying degrees, most decision analyses on the choice of antithrombotic therapy for atrial fibrillation are sensitive to patients utility for the inconvenience associated with warfarin administration [13-17], which has been as low as 0.92 on a 0 to 1.0 scale where 0 is death, and 1.0 full health [18]. Failure to present all the inconveniences of warfarin use in vivid detail ("unpacked" and described in its many components) could lead to an underestimation of its relative importance [19].

Second, we did not include consideration of deaths from all cause and those resulting from bleeding. The literature does not emphasize the 20% reduction in the risk of dying associated with warfarin when compared to placebo [7]. There are fewer deaths (although this difference does not reach statistical significance) among patients using warfarin than among patients using aspirin [11]. However, as the bleeding risk increases with warfarin (as we will model during the probability trade-off exercise) the number of deaths due to bleeding (at most, 10% of severe gastrointestinal bleeds will die) will also increase and eventually offset the small probable mortality benefit of warfarin over aspirin. We instructed clinicians and patients to consider the scenarios as presented, ignoring other issues that might come into play in other situation. It is possible, however, that while patients were easily able to follow these instructions, clinicians incorporated their knowledge of issues of inconvenience of warfarin use and of bleeding deaths in their evaluation.

Limitations of our prior study, however, permit alternative explanations of our findings. In particular, we did not highlight for patients the inconvenience of warfarin therapy, the risk of death with bleeding, nor the risk of death with strokes. Discrepancies in clinician and patient responses may be explained by clinicians taking these factors into account, while patients did not. The goal of this proposal is to resolve uncertainty in the interpretation of our prior study, and deepen our understanding of how clinicians make decisions about warfarin use in patients with atrial fibrillation. The implications of the study proposed are potentially profound: if differences in clinician and patient values and preferences underlie the current apparent underutilization of warfarin, it can be inferred that there are truly hundreds of thousands of patients experiencing an unnecessary devastating stroke each year. Such a result will mandate the development and testing of interventions to align clinician behavior with patient preferences.

## Design

The proposed research seeks to answer the following questions:

1) When assessed systematically, do patients' and clinicians' preferences explain the utilization of warfarin to prevent strokes associated with atrial fibrillation?

2) To what extent do patients' and clinicians' treatment preferences differ?

3) What explains any differences in treatment preferences between patients and clinicians?

To answer these questions we will conduct a two-phase study of patient and clinician preferences for health states and treatments. In the first phase of this study we will conduct structured interviews with patients and clinicians to determine their treatment preferences for warfarin vs. aspirin treatment to prevent strokes associated with atrial fibrillation. In the second phase of the study we will convene focus groups of patients and clinicians to explore their answers to the exercises in the first phase. The ethics committee of the Hospital Sant Pau (Barcelona, Spain) approved the study.

### Setting and target population

#### Setting

We will conduct this study at three cities in Spain (Barcelona, La Coruña and San Sebastian). For each site several hospitals will collaborate to recruit the required number of cardiologists and internists. For the recruitment of general practicioners we will recruit from the Primary Health Care areas that belong to these hospitals in each of the three cities. This multicenter approach will limit the influence of local views about treatment of atrial fibrillation on the results of our study and will increase generalizability.

#### Patients

We will enroll patients 60 years of age or older with one or more of the following conditions: diabetes, hypertension and history of cardiac disease (heart failure and myocardial infarction). Exclusion criteria include: mini-mental state examination (MMSE) [20] score < 24, inability to complete the research tasks, history of any form of atrial fibrillation, history of using warfarin (but not of using aspirin or any other anti-platelet agent since their use is ubiquitous in the target population), inability to participate because of illness, or unavailability.

Patients with newly diagnosed atrial fibrillation who are contemplating the choice of antithrombotic therapy to prevent strokes represent the ideal patient population. There are, however, logistical challenges in prospectively identifying such a group and in ensuring their participation prior to their making a decision about using or not using antithrombotic therapy. Not enrolling patients after they have made a decision regarding anticoagulation is crucial to prevent bias as a result of cognitive dissonance. Cognitive dissonance (a state of psychological discomfort due to inconsistent cognitions) could readily lead patients to modify their interpretation of information provided during the study to ensure it was consistent with their previous decision, a decision that may or may not have been well-informed [21].

The next best choice is to enroll patients at high risk of developing atrial fibrillation who may have to make this choice in the near future. This proximity to the choice increases the likelihood that patients will view the exercise as relevant to them and we will obtain meaningful responses that are reflective of the patients' true preferences. Thus, the eligible patient will be at high risk of atrial fibrillation according to data from the Framingham study [6]. This study identified the following risk factors for atrial fibrillation: age, hypertension, diabetes, history of cardiovascular disease, valvular heart disease, and heart failure. These inclusion and exclusion criteria are broader than but consistent with eligibility criteria used in our previous work in which we recruited patients with previous myocardial infarction or heart failure [12].

#### Recruitment strategy

We will use several strategies to recruit patients and clinicians for this study. In the case of patients we will sample them from the different databases of the Health Areas from the three sites where the study will take place. Each site will provide a random sample of patients fulfilling eligibility criteria for participation. We will invite these patients to participate initially with a phonecall from their primary care physician or a delegate from their center. The study coordinator will follow-up with a phone call to verify eligibility, obtain verbal consent, and set up an interview date. Patients will give written informed consent to participate.

We will sample practicing clinicians working in general medicine (primary care, family medicine, internal medicine) and in cardiology, based in the community or in the hospitals. Clinicians who spend less than 30% of their time seeing patients in the outpatient or inpatient settings or if they have not cared for a patient with atrial fibrillation in the preceding six months are ineligible to participate. These eligibility and exclusion criteria should select clinicians who participate in making decisions about antithrombotic therapy with patients with atrial fibrillation, the most relevant group for our study. We will send an electronic letter to clinicians inviting them to participate in this study. A second mailing will follow if the local investigator receives no answer. Finally, clinicians who have not responded to the 2 mailings will receive a phone call from the local study investigator (a local colleague) inviting them to enroll.

In our experience, time is the main barrier to clinician participation. We plan to complete each clinician interview within 30 minutes. To achieve this goal, we will use a highly scripted interview with extreme economy of items. To ensure the success of the focus groups, we will use multiple ways to get clinicians to the focus group venue on time. These include, but are not limited to, sending periodic e-mail reminders, contacting their administrative assistants to include the focus group on their daily calendars, making same-day reminder phone calls, and arranging for transportation to the focus group venue, as needed.

### First phase: the individual interview

The clinical context of this decision study is the use of antithrombotic agents to treat chronic non-valvular atrial fibrillation. Both patients and clinicians will go through an individual highly scripted interview. To determine their strength of preference for use of antithrombotic agents we will conduct a probability trade-off exercise. This will answer the first research question and will allow us to determine if there is a difference and if present the magnitude of the difference in strength of preference for treatment between patients and clinicians, the second research question.

To partially answer the third research question (whether differences in utilities for the relevant health states explain differences in treatment preferences) we will ask participants to assign relative value to the relevant health states using a visual analog scale (also known as the feeling thermometer). The complete elicitation of treatment preferences and utilities will take approximately 15 minutes. The patients will receive the mini-mental state examination and 2 additional "screening" scenarios to gauge their ability to participate in the probability trade-off exercise. Thus, we expect patient interviews to take 45 minutes and clinician interviews to take 30 minutes to complete.

To account for order effects, we will randomize the order in which participants complete the exercises (probability trade-off and feeling thermometer) and the health states within the feeling thermometer (mild stroke, major stroke, taking warfarin, taking aspirin, severe bleeding). Clinicians could answer questions thinking about what they would choose for themselves (if they were to have atrial fibrillation) or what they would recommend to their patients. To eliminate this ambiguity (and study this potential effect), and following the method by Cohen and Pauker [22], we will randomize clinicians to instructions that ask them to participate and complete the preference elicitation tasks in the role of a patient or as a clinician making a recommendation to a patient.

Prior to the preferences elicitation exercises, patients will complete a brief questionnaire indicating their age and gender and whether they have personal knowledge of someone who had a major or a minor stroke, or a major gastrointestinal bleed. Likewise, clinicians will indicate their demographic characteristics, the number of years in practice, specialty, and experience with atrial fibrillation, bleeds and strokes.

#### The health states

The decision to use antithrombotic agents in patients with atrial fibrillation at risk of stroke involves a choice between aspirin or warfarin use. "No therapy" is a realistic option only for the lowest risk patients, or those with a serious bleeding problem. Decision analyses [23] and decision support [24] investigators have shown that the choice of antithrombotic therapy is sensitive to preferences for the following health states: major and minor stroke, major gastrointestinal bleed, and taking either warfarin or aspirin daily (including the need for periodic monitoring and lifestyle modification with warfarin). We plan to use the same health state descriptions for stroke and major bleed that we used in a previous study [12], which in turn were modified from Man-Son-Hing et al [25]. These are almost identical to other descriptions in the literature and have three advantages: (1) have been published before and were deemed valid to peer investigators in the field of decision making; (2) have been used with clinicians for the conduct of a decision study and therefore were deemed valid and understandable to participants similar to those we propose to enroll for the present study; (3) their use will allow our results to compare to other studies in this field, including our previous study in a different sample of clinicians.

#### Probability trade-off

**Figure 2:**
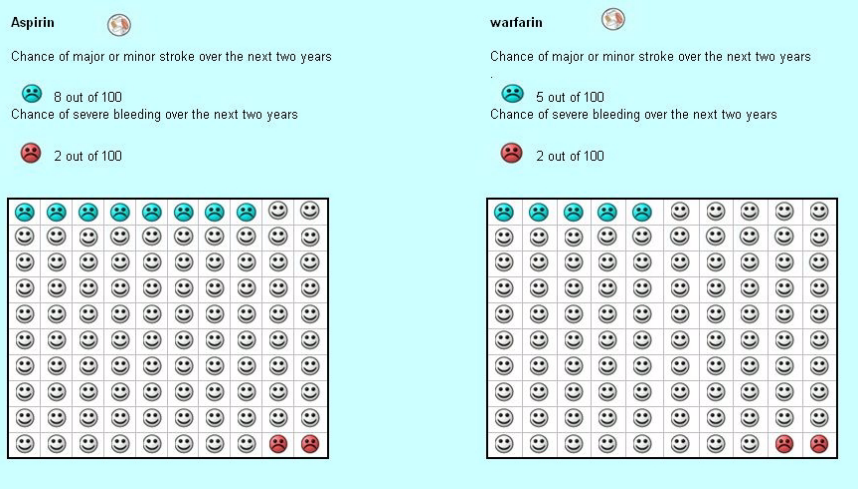
**Section of the decision board for probability trade-off**[1]**comparing outcomes of2 years of warfarin usevs. aspirin among 100 patients with atrial fibrillation, as used in our preliminary study**[12].

We propose to use the probability trade-off technique to determine the strength of preference patients and clinicians have for antithrombotic use to prevent strokes associated with atrial fibrillation. We have described the conduct of the probability trade-off exercise for the same decision context [12]. During the interview, the researcher, following a pre-written script, presents information both verbally and visually with the use of colored pictorial flip charts, known as decision boards (Figure 2). Participants will review the decision boards describing major and minor stroke, major bleeding, and inconveniences and costs of treatments. After presenting the descriptions, interviewers will ask participants if the scenarios reflect their own appreciation of the issues involved in the decision and will note any discrepancies. Whatever their response, we will ask participants to provide responses assuming that the scenarios represent an accurate and complete characterization of the health states. This instruction seeks to reduce information asymmetry between patients and clinicians and between specialists and generalists. Also, we will use the same decision boards and the same scripts to interview both patients and clinicians. The only difference in the scripts is that clinicians, when randomized to respond like a clinician making a recommendation to a patient, would need to indicate whether they would recommend using warfarin at the given risks while patients would need to indicate whether they would *use *warfarin at the given risks.

During the individual interview, we will present complete information about the risks, benefits, and inconveniences of warfarin and aspirin. To demonstrate how we will determine a patient's threshold for accepting a treatment we will discuss the scenario for determining the maximum bleeding increase acceptable for warfarin therapy (i.e. the bleeding threshold for warfarin) in a comparision of warfarin vs. aspirin.

The scenario is based on the absolute effects of both drugs on 100 patients over a two-year period. We will use a probability trade-off with the elicitation method of "ping-ponging" to determine participant's thresholds. The interviewer will systematically vary the risk of bleeding with warfarin (alternating between high and low risk of bleeding) to determine the maximum acceptable increase in the risk of bleeding with warfarin, given a fixed reduction in the risk of stroke with warfarin relative to a fixed risk of bleeding and stroke with aspirin. The alternating presentation of high and low risk of bleeding may reduce "start point bias" resulting from starting the exercise at the point of known efficacy and safety of the medications.

Given the risk of bleeding with aspirin is two patients in a hundred (over a two year period) we will offer probabilities from 0 additional bleeds with warfarin to 38 additional bleeds with the use of two sets of flipcharts (one exploring up to 17 and one exploring up to 38 additional bleedings). In our previous study, we compared warfarin versus no treatment and the mode for patients' bleeding threshold was 22 additional bleeds per 8 fewer strokes with warfarin therapy (10.3 [6.1] and 17.4 [7.1] additional bleeds for physicians and patients respectively) [12]. The 22 additional bleeds corresponded to the maximum bleeding threshold explored. Since in this proposal the number of strokes avoided is 3 (in the comparison of warfarin versus aspirin), we will increase the range explored by going up to 38 additional bleeds. Additionally, in the case of participants willing to take warfarin given an increased risk of an additional 38 bleeds, we will ask them, without the use of flipcharts, what is the maximum number of bleeds they would accept before switching to aspirin (being 100 the maximum). This range should ensure capturing the preferences of all clinicians and most patients interviewed.

Initially, participants will complete two eligibility scenarios that compare the outcomes of stroke and bleeding with two ficticious drugs with the same adverse effects, burdens and cost, but with major differences in terms of stroke reduction. If participants do not select the medication with the higher stroke reduction in both instances they will be excluded as their choice reflects inadequate comprehension.

The decision boards will reflect the best available evidence, namely from the meta-analysis of warfarin vs. aspirin for atrial fibrillation [11] (Table 1). Our scenario will start with a statement of the baseline risk–"with aspirin treatment there is a baseline risk of a major or minor stroke over the next two years of 8 patients out of 100 and a risk of severe bleeding over the next two years of two patients out of 100. In the case of warfarin there is a baseline risk of major or minor stroke over the next two years of 5 patients out of 100". We will then present that risk of bleeding using a flipchart. In this first flipchart we will start randomly with either no additional bleedings or with up to 17 additional bleeds. If the participant decides that she would still take warfarin with 17 additional bleeds, with the help of a second flipchart, we will offer up to 38 additional bleeds (starting either at 18 additional bleeds or at 38 and using the same "ping-ponging" method). In the case of a patient or physician accepting the 38 additional bleeds we will ask her what is the maximum number of bleeds she would accept before switching to aspirin.

**Table 1:** Summary of results of meta-analysis of warfarin vs. aspirin in patients with atrial fibrillation [11]

	**Risk % for 2 years**		
	Aspirin*	Warfarin	Difference between aspirin and warfarin

Stroke			

All stroke	7.8	5.0	2.8
Ischemic stroke	7.4	4	3.4
Fatal ischemic stroke	0.925	0.5	0.425
Hemorrhagic stroke	0.4	1.0	-0.6
Fatal hemorrhagic stroke	0.19	0.35	-0.16
Total stroke deaths	1.15	0.85	0.3
All deaths**	10.2	9.4	0.8

Bleeds			

EC bleeding***	2	3.4	-1.4
Fatal EC bleeding	0.175	0.32	-0.145

*, Data correspond to the aspirin alone arms with exclusion of the aspirin + low-dose warfarin arms of the pooled studies; **, including stroke deaths, cardiovascular deaths, but not extracranial bleeding deaths; ***, EC = extracranial, mostly severe gastrointestinal bleeds requiring admission to hospital, 2 units of blood, or surgery.

We will present patients and clinicians with the choice between warfarin and aspirin use as we think that is the dominant choice for patients with atrial fibrillation at average risk of stroke. For a group, perhaps as large as 20% of patients with atrial fibrillation (i.e., young patients without hypertension, diabetes, or previous stroke) the pertinent choice may be between aspirin and no antithrombotic therapy [26]. This group will not be represented in our intended sample of patients (i.e., individuals at risk of atrial fibrillation > 60 years-old, with hypertension, diabetes, or previous cardiovascular events). Adding this choice (or the choice of warfarin vs. no antithrombotic therapy) to the probability trade-off would require more time than feasible during clinician interviews. More importantly, the choice between warfarin and aspirin is most likely to demonstrate omission bias (physicians may place a relatively higher value on avoiding a bleed than on avoiding a stroke because they do not witness the strokes they have prevented) or differences in values and preferences between patients and clinicians, if these were present. Therefore, we will limit the probability trade-off exercises to the choice of warfarin vs. aspirin.

#### The feeling thermometer

To further understand the treatment preferences elicited using the probability trade-off and capture the values patients and clinicians place on the health states that may result from their choice, namely strokes and bleeds, we will conduct preference-elicitation exercises using the feeling thermometer, a visual analogue scale [27,28]. When completing the feeling thermometer patients choose the score on the thermometer that represents the value they place on the health state they are being asked to evaluate. The feeling thermometer is anchored at death (0) and perfect health (100). The feeling thermometer will allow us to check understanding of the relative severity of the health states in question (i.e., patients should assign a lower value (a value closer to 0) to a major stroke with severe and permanent disability than a minor stroke).

Because the visual analogue scales may be prone to context bias, that is rating of one state may influence rating of other states [29,30], we will randomize the order in which participants will consider the health states to minimize this bias. We will ask participants to consider the following health states one at a time on the feeling thermometer:

▪ Major and minor strokes. We will inform patients that the sequelae of the stroke will last for the patient's life expectancy.

▪ Severe bleed. We will inform patients that they will have a major bleed within the first year of taking the antithrombotic agent but will not have another severe bleeding event for the rest of their life

▪ Taking warfarin

▪ Taking aspirin

### The second phase: the focus groups

Qualitative research methods are well suited to study personal meaning, perceptions, beliefs, and values [31]. These methods are therefore appropriate to provide clinicians and patients with the opportunity to make explicit the thinking process, and explore the various hypotheses suggested as to why participants hold the views they do. Individual in-depth interviews and focus groups could provide this information. The individual clinician interviews will create an appropriate mindset for participants to consider in the focus groups *why *they hold the views they do. An important advantage of focus groups over in-depth personal interviews is the opportunity to explore dynamic interactions among the group members. These interactions include sharing of ideas and insights, stimulating others to consider the issues that may underlie their own preferences, and exploring areas of consensus and dissensus [32].

Thus, the focus groups are most likely to offer plausible explanations of clinicians' and patients treatment choices and the reasons for any inconsistency of these choices with their utilities for the health states of interest. If our results of the first phase confirm our preliminary findings, then these explanations will shed light on differences in preferences between clinicians and patients.

#### Setting and participants

At each of the 3 sites we will convene focus groups of clinicians and patients. We will follow the recommendation to keep the focus groups as homogeneous as possible in terms of preferences regarding the bleeding threshold [32]. To the extent that group members perceive others around the table as similar to themselves, they may feel safe to express themselves while expecting understanding and respect from the other members. We plan to convene six focus groups in total (3 of clinicians and 3 of patients). These numbers relate to our experience with focus groups and their information yield and to what is feasible for this proposal. Members of the focus group will be recruited from phase I to represent different preferences

#### Group configuration and data collection

Each group will have 10 participants per session, a trained facilitator (a clinician) and an expert assistant (not a clinician) [32]. The facilitator leads the focus group, facilitates the group ensuring that all participate, ensures that the group completes the agenda on time, and monitors and manages the group dynamic. The assistant (i.e., an expert qualitative researcher) will participate in all focus groups at all site and will document seating arrangements, the first words of each member, take notes on interaction dynamics and nonverbal behaviors, and operate the tape recorders. A digital tape recorder (and a backup) equipped with tabletop microphones will document all acoustic data for later transcription. The setting will be a small conference room in the hospital or primary care center. We will pay particular attention to issues related to noise, will limit interruptions once the focus group dynamic begins, and will keep the meeting on time. Participants will spend about 90 minutes discussing the agenda described in the following section.

#### Focus group agenda

The goals of the focus group are to educate the members about the results of the individual interviews, and to explore why clinicians and patients have the preferences they do. The facilitator will first ask about this in an open ended way, allowing participants to express their views in their own words in order to explore what reasons they give. Another goal of the focus groups is to get members to make explicit their thinking process that led to those preferences, and to explore the various hypotheses suggested (e.g., omission bias, avoidance of clinician-associated risk) as to why clinicians hold the views they do. The facilitator will start the proceedings by setting ground rules, making introductions, and using warm-up exercises to set a comfortable and open environment for exchange of ideas.

To achieve the research goals of the focus group, the facilitator will guide focus group members through a structured process: The facilitator will first present results from the individual interviews. Aggregate data will be presented and in addition, each participant will receive the results of their individual responses during the preference elicitation exercises. The facilitator will then elicit member responses to open-ended questions that he will pose to the group about these findings. For example, the facilitator will check that group members understand the findings before further discussion. Then, the facilitator will ask whether group members are surprised about the findings and if so, why or why not. They will be asked to comment on why they hold the particular treatment preferences expressed in the individual exercises. This will be followed with more structured elicitation exercises. The facilitator will contrast the results of the treatment preference exercise with the results of the feeling thermometer exercises to the extent that these exercises reveal, on average, different apparent values placed upon the health states. In addition, the following contrasts will be presented to initiate or continue discussion with the group:

▪ Direct contrast of the choices and strength of preferences for antithrombotic treatment of the average clinician in the focus group and of the average patient in the study (i.e., the number of additional bleeds that clinicians and patients are willing to tolerate to prevent an additional stroke at the point of indifference between using warfarin and using aspirin).

▪ Direct contrast of the average utilities for stroke, bleeding, and using antithrombotic agents between patients and clinicians.

To promote and focus debate the facilitator may ask the following questions to the group (if appropriate given the results):

▪ Can you describe, in your own words, the thought processes you went through in making your decisions during the probability trade-off and feeling thermomether exercises?

▪ Do you think that your answers to probability trade-off and feeling thermomether are consistent with each other? Why or why not?

▪ Which one of the exercises (probability trade-off, feeling thermomether) do you think captured more accurately the true relative values you place on avoiding bleeds and preventing strokes? Why?

Depending on the results during phase 1 (individual interviews), other questions could be relevant, including the following:

▪ You placed a relatively higher value on bleeding and a relatively lower value on stroke when making a decision (probability trade-off) than when we looked at those outcomes separately (feeling thermomether). Why?

▪ Can you think of plausible explanations for the differences between clinicians and patients choices and preferences for treatment?

▪ Can you think of plausible explanations for the differences between the answers that clinicians gave when they participated as patients and when they participated as clinicians?

### Analysis plan

#### Research question 1

When assessed systematically, do patients' and clinicians' preferences explain the apparent underutilization of warfarin to prevent strokes associated with atrial fibrillation?

##### Description of treatment preferences

We will use probability trade-off results to determine the number of additional bleeds that participants are willing to tolerate per stroke prevented with warfarin at the point of switch to aspirin. We will call this variable *b*. We will describe the distribution of *b *in clinicians grouped by age, gender, years in practice, specialty, experience with atrial fibrillation, experience with bleeds and strokes, and whether they participated as clinicians or as patients. We will also describe *b *in patients grouped by age and gender, and by personal knowledge of someone who had a major or a minor stroke, or a major gastrointestinal bleed. We will also describe the distribution of *b *in clinicians and patients by site. We will conduct exploratory linear regression analysis using *b *as the dependent variable and clinician characteristics listed above as the independent variables. The results of this analysis will guide our choices for subgroup analyses to answer Research Question 2.

#### Research question 2

To what extent do patients' and clinicians' treatment preferences differ?

##### Differences in treatment preferences between patients and clinicians

We will test the difference of the means of *b *between the patients (*b*_*p*_) and clinicians (*b*_*c*_) using an unpaired *t *test. The observed distribution of *b*_*p *_in our preliminary work was highly skewed. This was because patients clustered at the highest *b *offered. For this study, we propose to offer a maximum *b *that is twice as large as the maximum *b *offered in our preliminary study. Furthermore, we will be studying a relatively large number of patients and clinicians with distribution of the means approximating the normal distribution with SEM = SD/vn. Our sample sizes will be considerably larger than 30, a size which is often taken as a guideline threshold, above which the sample distribution of the sample mean will be reasonably normally distributed, even if the distribution of individual values is substantially skewed. Thus, the use of the t-test is appropriate. However, we will also use the Mann-Whitney test, a non-parametric test that does not require the assumption of normality of the distribution. After identifying pertinent subgroups in the analysis to Research Question 1, we will conduct modified *t *tests to test the difference of the means of *b *between the patients and the subgroup of clinicians of interest. A modified *t *test is based on the pooled estimate of variance from all the subgroups [33]. The resulting *P *values can be adjusted for multiple comparisons using the Bonferroni method (multiplying the resulting *P *value by the number of paired comparisons performed).

We will also determine the proportion of patients and clinicians who would choose to take or recommend warfarin when b = 0.5 (that is, when patients experience one bleed for every two strokes prevented). We designate this value of b as *b*_*RCT *_because it is the average number of additional bleeds per stroke prevented reported in randomized trials of warfarin vs. aspirin from the meta-analysis [11] (Table 1). We will test the difference in these proportions using Fisher's exact test and will estimate the exact 95% confidence interval of the odds ratio for the proportions of patients and clinicians who would use warfarin at *b*_*RCT*_. We will also determine the proportion of clinicians from pertinent subgroups (identified in Research Question 1) who would choose to recommend warfarin at *b*_*RCT *_= 0.5. Then, we will test the differences between this proportion and the proportion of clinicians not in the subgroup recommending warfarin when *b *= 0.5, as well as the difference between the proportion recommending in the subgroup and the proportion of patients who would choose to take warfarin at *b*_*RCT*_.

#### Research question 3

What explains any differences in treatment preferences between patients and clinicians?

##### Differences in utilities between patients and clinicians

In responding to the feeling thermometer, each participant will provide 5 utilities (for major and minor stroke, major bleed, using daily aspirin, using daily warfarin). We will compare the distributions of each of these utilities in patients and clinicians using the Mann-Whitney test, a nonparametric test, as these variables are usually highly skewed. We will also use the *t *test to test the difference of the means of the utilities between patients and clinicians. To evaluate the consistency between participant utilities for health states and *b *(that is, the number of additional bleeds per stroke prevented at the point of switch from warfarin to aspirin in the probability trade-off exercise) we will examine scatter plots of each of the utilities and *b*. Also, we will estimate the correlations between each of the utilities for the 5 health states and *b*. We will conduct linear regression analysis with *b *as dependent variable and with utilities as independent variables. We will attend to the interaction between the utilities, which we anticipate being a key issue, in this analysis.

##### Analysis of focus group data

Data from the 6 focus groups sessions will be transcribed verbatim and checked for accuracy by comparing the written text to each audio tape. Initially, two experienced researchers in qualitative methods using an editing approach [34] will work independently to develop a coding system to identify the major themes from data contained in two of the focus groups transcripts. The researchers will then meet to compare the consistency and meaning of the codes developed and their application in the first set of two transcripts. Based on these results, researchers will then modify the coding scheme based and establish an audit trail [35]. Then, the two researchers, working independently, will code two further focus group transcripts using the coding scheme and again compare results, discussing and resolving any disagreements. This iterative process will continue until researchers finish coding all the transcripts. The codes (themes) developed will focus on capturing the kinds of thought processes and rationales that clinicians used to make their treatment preferences in the first phase interviews. In the focus group, clinicians will receive feedback on the results of the first phase interviews to set the context for the discussion of reasons for these preferences. The results will include the distribution of preferences of patients and clinicians, differences in these distributions, and the degree of internal consistency between clinicians' treatment preferences and their utilities for the relevant health states.

Data from focus groups transcripts will be entered into Atlas.ti , a computer friedly software program that assists with data management, search, and retrieval of textual information [35]. Data analysis will occur at three different levels: description, analysis, and interpretation [36]. In terms of description, we will develop a structured form to summarize key information from each focus group transcript. We will also describe data segments from the transcripts and code them by theme. Next, we will undertake a comparative a nalysis of themes across the six focus groups as a whole to identify similarities and differences in the data. Finally, we will identify how participants think about the underlying thought processes they used to determine their preferences and point out implications for practice. During the data analysis and write up stages, we will ask clinicians from each focus group to review and provide feedback on the written summary prepared for their specific group in order to ensure that these summaries accurately reflect the key themes from the participants' perspective [32,37,38].

##### Sample size estimation

We require six focus groups, 1 of generalists and 2 of cardiologists at Barcelona, 1 of generalist at Galicia and País Vasco. We will undertake 1 group of patiens in each site. Ideally, each group should have 8 to 10 participants. We anticipate that not every clinician will be able to participate. To ensure 8 clinicians per group, we will enroll 12 potential participants per group. We will construct the focus groups to maximize homogeneity of specialty, experience with patients with atrial fibrillation, and treatment preferences during individual interviews (phase 1). From our survey of faculty at the 3 sites, less than 10% of clinicians at each site are not eligible. Therefore we would need to enroll 20% of eligible clinicians at each site to achieve our sample size of 96 clinicians which will ensure adequate numbers for the focus groups.

Our study should be powered to determine differences in means of *b*_*p *_and *b*_*c*_, that is, differences in the treatment preferences of patients and clinicians, the key variable. In our previous study, the effect size (difference in mean *b *between patients and clinicians) was 0.9 (this is calculated from sample means (and SDs) of 2.2 (0.9) and 1.3 (0.8) in the patients and clinicians, respectively). Although it is not immediately evident what magnitude of the difference in *b *would be clinically important, readers and commentators have uniformly considered the differences we showed as important. Table 2 shows the power we would have with the proposed sample size of 96 clinicians and an equal number of patients, and also illustrates the limited impact of increasing the number of patients from 1:1 to 1:3. Therefore, given the number of clinicians available to us, we will have ≥ 80% power to detect differences in means ≥ 0.4·SD. We will have ≥ 80% power to detect differences in means of *b*_*p *_and *b*_*c *_≥ 0.75·SD between subgroups of ≥ 20 clinicians and 96 patients. In summary, after enrolling 96 patients and 96 clinicians we will have ≥ 80% power to detect (1) *b*_*p *_- *b*_*c *_> 0.4 SD, (2) odds ratio ≥ 2.6 of the proportion of patients and clinicians with *b *> *b*_*RCT *_(if that proportion is 0.63 or smaller among clinicians), and (3) a difference in mean utilities of ≥ 0.4 SD. We need to enroll 96 clinicians to ensure 8 members per focus group. Enrolling more than 96 patients has a very small impact on power. Thus, we will recruit 96 patients and 96 clinicians for this study.

**Table 2:** Power to detect differences in means of *b*_*p *_and *b*_*c *_0.2 to 0.5 standard deviation units (α = 0.05)

***Sample***		*b*_*p *_- *b*_*c *_difference in SD units			
Clinicians	Patients	0.2	0.3	0.4	0.5
96	96	.28	.54	.79	.93
96	144	.33	.62	.86	.97
96	192	.36	.67	.89	.98
96	288	.39	,71	.92	.99

## Discussion

Thousands of patients with atrial fibrillation will suffer preventable strokes this year because they do not receive anticoagulation. A number of lines of evidence, including our own prior study, suggest that differences between clinician and patient preferences regarding warfarin versus alternative management of atrial fibrillation may explain this apparent underutilization of anticoagulation. If this is so, development and testing of interventions to increase rates of anticoagulation in patients with atrial fibrillation should become a matter of urgency.

The evidence is not, however, strong enough to justify proceeding confidently with the development and testing of such interventions. In particular, we have identified methodological limitations in our prior work that raise the possibility that a different understanding of the options presented may explain differences in clinicians and patients choice. We now propose to repeat our prior study with a stronger methodology that will allow us to determine the true explanation of the results. We may find that, when they understand the options in a similar way, clinicians and patients make similar choices. If this proves to be the case, it would suggest that the low rates of anticoagulation in patients with atrial fibrillation reflect a shared aversion to bleeding risk in clinicians and patients. Such a finding would prompt the conclusion that current rates of anticoagulation are, in fact, appropriate.

Our review of the evidence suggests that it is more likely that we will confirm substantially different treatment choices in clinicians and patients. This result would suggest that current low rates of anticoagulation in atrial fibrillation reflect clinician and not patient values, and mandate urgent development and testing of interventions to increase rates of anticoagulation, and thus prevent unnecessary strokes. Such findings would not, however, explain why clinician and patient choices differ. We see two leading possibilities. One is that, relative to patients, clinicians experience the post-stroke state as relatively less aversive, and having a gastrointestinal bleed as relatively more aversive, than do patients. The second explanation is that clinicians and patients share the same values for stroke and bleeding, but the circumstances of clinical decision-making lead clinicians to make different choices. In particular, we hypothesize that omission bias associated with efforts to reduce clinician-generated risk, may explain the results. This explanation posits that clinicians put more weight on commission of a behavior that leads to adverse consequences (bleeding through warfarin administration) than an omission of behavior that could lead to beneficial consequences (stroke prevention through warfarin administration). We will explore this issue through direct measurement of utilities for stroke and bleeding, and through focus groups with the participating clinicians.

On completion of this study, we will hope to achieve major advances in understanding clinician and patient decision-making in atrial fibrillation. Our anticipated results will challenge investigators, medical educators, and health care providers to develop and test strategies to deal with differences in clinician and patient choices. Our results will guide the development of these strategies, which may include innovations in undergraduate and post-graduate education, enhancements to the clinical encounter to facilitate patient-clinician communication and shared decision-making, increasing use of decision support tools in clinical practice, and monitoring and feedback of clinician success in providing optimal anticoagulation in patients with atrial fibrillation. For example, our experience using decision boards in this study will inform the organization and conduct of a randomized controlled trial to test their efficacy in helping patients with atrial fibrillation make informed choices.

## Abbreviations

AF: atrial fibrillation; EC: extracranial; N: sample size; SD: standard deviation; SEM: Standard error of the mean.

## Competing interests

The authors declare that they have no competing interests.

## Authors' contributions

VM, GG, HS, PD, CC and PA-C participated in the conception and design of the protocol and drafted a first version. All authors participated in revising it critically for important intellectual content and have given final approval of the version to be published.

## Pre-publication history

The pre-publication history for this paper can be accessed here:


